# Improving the Sensory, Nutritional and Technological Profile of Conventional and Gluten-Free Pasta and Bakery Products

**DOI:** 10.3390/foods10050975

**Published:** 2021-04-29

**Authors:** Barbara Simonato

**Affiliations:** Department of Biotechnology, University of Verona, Strada Le Grazie 15, 37134 Verona, Italy; barbara.simonato@univr.it

There is currently a growing consumer interest in healthy food. Cereal-based products such as pasta and baked goods represent staple foods for human nutrition. Due to their worldwide diffusion, these products can be carriers of nutrients and bioactive compounds; therefore, they lend themselves very well to the fortification process. Furthermore, among new formulations of cereal-based food, gluten-free products have become popular even among people without celiac disease, who have chosen a gluten-free lifestyle. The improvement of well-being, sustainable lifestyles, and waste control are also aims of the United Nations for the Agenda 2030 (UN 2015), which has motivated food scientists and industrial producers to research new and healthier formulations for pasta and baked goods preparation. In this context, researchers are also encouraged to use agro-industrial by-products of high added value for food fortification. In this frame, the Special Issue “Improving the Sensory, Nutritional and Technological Profile of Conventional and Gluten-Free Pasta and Bakery Products” collected nine original articles focused on new product formulations of gluten-free pasta or baked products formulation, as well as agro-industrial by-product utilization. The final aim was the preparation of valuable products from a nutritional, technological, and sensory standpoint.

Cappa et al. [1] studied the effects of red rice or buckwheat flour inclusion in potato-based pasta (gnocchi) concerning the nutritional, technological, and sensory characteristics of the final product. The researchers concluded that gnocchi fortified with buckwheat flour showed better texturizing characteristics and could benefit from the claim “source of fibre”. Nevertheless, wholemeal buckwheat is responsible, due to its particle sizing, for a negative response from a sensory point of view, thus reducing the acceptability of the fortified gnocchi.

As part of the production of healthy foods, Krupa-Kozak et al. [2] produced a gluten-free sponge cake by replacing sucrose with fructans of different degrees of polymerization, to observe its technological and sensory characteristics. The authors demonstrated that sucrose is not necessary to obtain a gluten-free sponge cake with favorable technological and sensory features, as the one prepared with fructooligosaccharide achieved the highest score for overall quality acceptance at sensory analyses.

Belorio et al. [3] investigated the utilization of hydrocolloids as a possible approach to optimize the hydration level of gluten-free bread. The authors, evaluating the effect of different hydrocolloids on gluten-free bread’s textural aspects, concluded that a thorough investigation of the use of hydrocolloids and starch source mixtures would be required to optimize the gluten-free bread texture.

Arribas et al. [4] studied the effect of cooking on the bioactive compounds content, texture, and color properties of rice/bean-based pasta fortified with carob. The authors’ findings revealed that, also after cooking, the fortified pasta maintained its healthy characteristics thanks to the high amount of the detected bioactive compounds. Moreover, this gluten-free pasta did not show the antinutritional factors of bean flour and presented appreciable textural parameters.

Within the framework of gluten-free products, Yu et al. [5] highlighted the importance of gluten detection methods. Currently, several enzyme-linked immunosorbent assay (ELISA) tests are used to detect the trace of gluten, and in their research, the authors observed that three different ELISA test kits often returned values below the detection limits. The authors underlined the importance of developing an accurate analysis method for the detection of gluten traces.

Regarding the fortification process, Tolve et al. [6] enriched pasta with two different levels of grape pomace, an agro-industrial by-product rich in fiber and phenols. The researchers observed an improvement in pasta’s nutritional properties and a reduction of the predicted glycemic index. Grape pomace inclusion instigated changes in the cooking and textural properties of pasta. The final product had good overall acceptability from a sensorial point of view. Furthermore, the bread’s fortification with two different levels of grape pomace (Tolve et al., 2021) [7] provoked modifications in the rheological properties of the doughs and textural characteristics of the bread samples. The grape pomace inclusion gave rise to the more tenacious and less extensible dough and bread with lower volume. Nevertheless, bread fortification improved the nutritional properties, increasing the total phenol content and the antioxidant capability. The bread samples showed good overall acceptability. 

The findings reported in the last two articles suggest that grape pomace represents an interesting ingredient for pasta and baked food fortification, due to the high content in phenols and dietary fiber.

Sissons et al. [8] investigated the amylose content, which is positively correlated with resistant starch, to lower the glycemic index of pasta produced by durum wheat (cv Svevo) by silencing the key genes involved in starch biosynthesis. The results showed that pasta obtained from durum wheat mutants had overall quality acceptability and the starch-branching enzyme IIa’s mutation provided a better glycemic response.

Pasini et al. [9] evaluated the technological properties of pasta production with semolina from cv Biensur, produced in zones with different fertility and treated with various rates of N, in comparison with commercial semolina (cv Aureo). The results obtained in this research demonstrated that the technological properties of Biensur semolina correlated to the low fertility zones treated with a high quantity of N. The derived pasta had characteristics similar to the ones obtained by semolina in cv Aureo. The higher amounts of gluten proteins, and the higher glutenin/gliadin ratio in semolina, represent good indexes of technological quality.
